# Myroides odoratimimus-Associated Skin and Soft Tissue Infection in Critical Limb Ischemia With Progressive Gangrene: An Emerging Environmental Pathogen

**DOI:** 10.7759/cureus.99138

**Published:** 2025-12-13

**Authors:** Jyothsna Goranti, Ricardo Olivas Lerma, Reyes Castanon, Jeffrey Sherwood

**Affiliations:** 1 Internal Medicine, Texas Tech University Health Sciences Center El Paso, El Paso, USA; 2 Internal Medicine, Paul L. Foster School of Medicine, El Paso, USA; 3 Infectious Disease, Texas Tech University Health Sciences Center El Paso, El Paso, USA

**Keywords:** antibiotic resistance, diabetes, environmental health, myroides odoratimimus, vascular disease, wound infection

## Abstract

*Myroides* species are rare but increasingly recognized opportunistic pathogens, primarily affecting immunocompromised individuals and those with environmental or nosocomial exposures. These organisms exhibit intrinsic multidrug resistance, making infections difficult to treat. We report a case of a 59-year-old woman with poorly controlled diabetes mellitus who presented with progressively worsening bilateral foot ulcers in the context of newly diagnosed severe peripheral arterial disease. Her history was notable for saltwater exposure in Acapulco, Mexico, and multiple prior hospital encounters. Wound cultures grew *Myroides odoratimimus* exhibiting high-level antimicrobial resistance. Despite broad-spectrum intravenous antibiotics and attempted revascularization, her infection and ischemia progressed, necessitating a below-knee amputation. This case underscores the emerging clinical relevance of *M. odoratimimus* as an environmental pathogen capable of causing destructive soft-tissue infections in patients with chronic comorbidities. It highlights the importance of early culture-guided antimicrobial therapy, recognition of potential environmental exposure sources, and prompt surgical intervention for infection control and limb salvage.

## Introduction

In 1996, genotypic and phenotypic analyses demonstrated that *Flavobacterium odoratum* was distinct from other members of its genus, prompting its reclassification into the novel genus *Myroides* [1]. Several species, including *Myroides odoratus* and *Myroides odoratimimus,* have been implicated in human disease.

*Myroides* are aerobic, yellow-pigmented, non-motile, Gram-negative rods that are ubiquitous in nature and act as opportunistic pathogens [2]. Although primarily associated with infections among immunocompromised hosts, recently published case reports have also described *Myroides* infections in immunocompetent patients [2,3]. Soft-tissue and urinary tract infections are the most commonly reported, but cases of pneumonia, septic shock, endocarditis, and ventriculitis have also been linked to *Myroides* [4-6]. Successfully treating these infections can be challenging owing to *Myroides*’ capacity for biofilm formation and its extensive antimicrobial resistance profile [7,8]. Case reports and genomic analyses of *Myroides* have demonstrated broad resistance to β-lactams coupled with varying susceptibility to aminoglycosides, fluoroquinolones, and trimethoprim-sulfamethoxazole [2,7-11]. Additionally, both a cohort study and a large-scale genomic analysis of antimicrobial susceptibility profiles have demonstrated sensitivity to meropenem as a treatment option [10,11].

*M. odoratus* and *M. odoratimimus* have been reported to naturally inhabit soil and freshwater environments [2,3]. Nosocomial outbreaks of *Myroides* have been traced to contaminated water sources, including a documented episode of catheter-associated bloodstream infections linked to ampules containing *M. odoratus* [2,12]. Other *Myroides* species have been isolated from a wide range of environments, including seawater, deep-sea sediments, and human saliva and urine [2,9]. Identified risk factors for infection from these organisms include having indwelling catheters or devices, prolonged hospitalization, invasive or surgical procedures, diabetes, and chronic kidney disease [6].

We present the case of a 59-year-old female with poorly controlled diabetes and a history of exposure to saltwater and multiple hospitals for wound care purposes who developed progressively worsening necrotic foot wounds in the setting of concomitant limb ischemia and *M. odoratimimus* wound infection.

## Case presentation

A 59-year-old Hispanic woman with a past medical history of type 2 diabetes mellitus complicated by peripheral neuropathy, hypertension, and osteoarthritis was brought to our emergency department by Border Patrol agents from a local detention center for evaluation of worsening bilateral foot ulcers. Three weeks prior, she developed small blisters on both feet that gradually enlarged and ulcerated. She reported soaking her feet in ocean water while traveling in Acapulco, Mexico, with the belief that this would help promote wound healing. She also described seeking care in hospitals in Mexico for her wounds, although specific details regarding topical wound care efforts or systemic antibiotic therapies were unknown. In the week leading up to her admission, she developed severe left foot pain associated with erythema, swelling, and foul-smelling purulent discharge. She also endorsed symptoms of paresthesias involving the soles of her feet. She had notably discontinued her diabetes medications (metformin and insulin) for well over a month due to a lack of access to prescriptions.

Upon arrival, she was afebrile but tachycardic. Examination revealed bilateral lower-extremity edema and ulcerations, with a dominant left lateral foot ulcer associated with drainage and cyanosis of the third and fourth digits. Dorsalis pedis and posterior tibial pulses were absent on the left. Laboratory studies were notable for leukocytosis and elevated inflammatory markers, along with a markedly elevated hemoglobin A1c of 14.4%. Renal function and liver enzyme levels were within normal limits, and blood cultures remained sterile. The detailed laboratory findings obtained on admission are summarized in Table 1.

**Table 1:** Laboratory findings on admission. BUN: blood urea nitrogen; ALT: alanine transaminase; AST: aspartate transaminase

Laboratory Test	Patient Value	Normal Range	Comment
White Blood Cell Count (WBC)	15.0 ×10^9^/L	4.0-10.0 ×10^9^/L	Elevated
Neutrophils (Segmented)	82%	40-75%	Elevated
Hemoglobin	11.4 g/dL	12-16 g/dL	Mild anemia
Platelet Count	348 ×10^9^/L	150-400 ×10^9^/L	Normal
Erythrocyte Sedimentation Rate (ESR)	92 mm/hr	<20 mm/hr	Markedly elevated
C‑Reactive Protein (CRP)	13.01 mg/dL	<1 mg/dL	Elevated
Blood Glucose	302 mg/dL	70-110 mg/dL	Hyperglycemia
Hemoglobin A1c	14.4%	<5.7%	Very poor glycemic control
Renal Function (BUN/Creatinine)	Normal	Normal	-
Liver Associated Enzymes (ALT, AST, Bilirubin)	Normal	Normal	-

**Figure 1:**
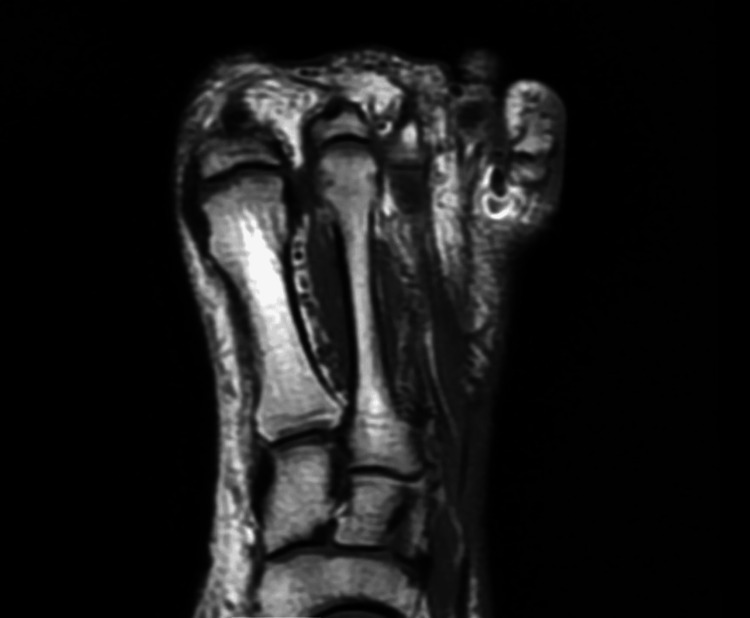
MRI of the left foot demonstrating soft-tissue edema. Coronal MRI of the left foot shows diffuse soft-tissue edema concerning for cellulitis. No evidence of abscess formation or osteomyelitis is identified.

Plain films of her left foot suggested evidence of soft tissue swelling without osseous abnormalities. MRI of her left foot noted soft tissue edema concerning for cellulitis, although no abscess or osteomyelitis was discovered (Figure 1). Bilateral lower extremity venous ultrasound was negative for deep vein thrombosis. Bilateral lower extremity arterial ultrasound confirmed severe peripheral arterial disease bilaterally, with absent flow in the posterior tibial, anterior tibial, and dorsalis pedis arteries on the left. She was admitted and placed on empiric broad-spectrum intravenous antibiotics in the form of piperacillin/tazobactam and vancomycin. The patient was started on piperacillin-tazobactam 3.375 g every eight hours and vancomycin 1750 mg every 24 hours on admission. During the course of her admission, a wound culture of left foot drainage was obtained, which yielded heavy growth of *M. odaratimimus,* demonstrating high-level drug resistance, as well as an isolate of *Providencia rettgeri* that was susceptible to most antibiotics reported. This prompted a transition to intravenous meropenem 1 g every eight hours. The antimicrobial susceptibility profile of *M. odoratimimus* is summarized in Table 2.

**Table 2:** Reported antimicrobial susceptibility profiles of Myroides odoratimimus. MIC: minimum inhibitory concentration; R: resistant; S: susceptible; I: intermediate

Antimicrobial Agent	MIC Interpretation	MIC (µg/mL)
Aztreonam	R	>16
Cefepime	R	>16
Ceftriaxone	R	>32
Ciprofloxacin	R	>2
Gentamicin	R	>8
Meropenem	S	≤1
Piperacillin/Tazobactam	I	32
Tobramycin	R	>8
Trimethoprim-Sulfamethoxazole (Bactrim)	S	2/38

**Figure 2:**
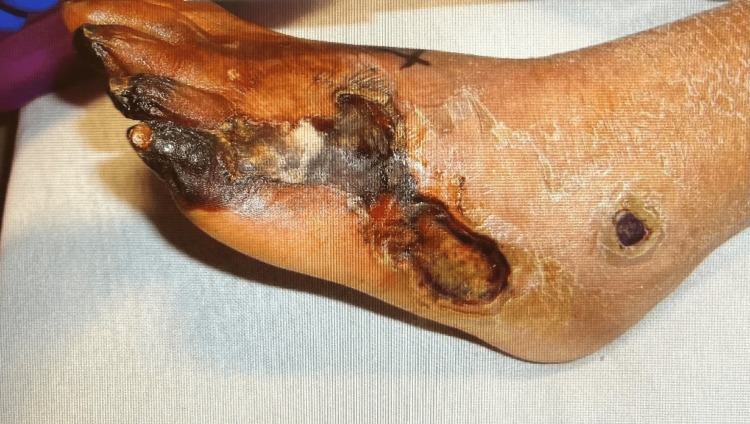
Photograph showing progressive gangrene of the left foot leading to amputation.

Glycemic control was achieved using a basal-bolus insulin regimen. Cardiology was consulted, and an angiogram was pursued, which confirmed complete occlusion of the left lower extremity arteries below the knee with a heavy clot burden. Balloon angioplasty failed to restore adequate flow on an initial attempt, and tissue plasminogen activator (tPA) was initiated. A second attempt at balloon angioplasty and aspiration thrombectomy restored partial flow in the anterior tibial artery, although no flow was achieved distally. The patient’s left foot gangrenous changes ultimately progressed, and the extremity was deemed unsalvageable, as demonstrated in Figure 2.

The patient agreed to a left below-knee amputation and underwent this procedure without complication. Viable bleeding tissue was noted at amputation margins without any residual gross evidence of infection. Postoperatively, antibiotics were discontinued, and pain control was achieved. The amputation site showed no evidence of infection, and her remaining right lower extremity wound was managed with local wound care. She was released on aspirin, clopidogrel, and a statin along with a diabetes management plan and analgesics. She was returned to law-enforcement custody for continued recovery and outpatient follow-up.

## Discussion

*M. odoratimimus* is an environmental Gram-negative bacterium that has emerged as an opportunistic cause of serious infection. It occurs naturally in soil and water and has been isolated from hospital water systems. Although considered a low-virulence organism, it is now recognized as a cause of invasive disease in patients with chronic comorbidities such as diabetes and vascular insufficiency [13,14]. This case illustrates how environmental exposure, repeated healthcare contacts, uncontrolled diabetes, and critical limb ischemia can intersect to produce a destructive, treatment-resistant infection.

Diabetes and advanced peripheral arterial disease create conditions that favor infection through impaired tissue perfusion and hypoxia. These changes weaken host defenses and reduce the ability of antibiotics to penetrate tissue. Our patient’s exposure to seawater during travel, followed by multiple hospital visits for wound care, created plausible environmental and healthcare sources for inoculation. Similar exposure patterns have been documented in outbreaks associated with contaminated hospital water and medical devices [15,16]. Ischemic tissue likely served as a protected focus where *Myroides* could persist and contribute to necrosis despite antibiotics.

Treatment of *Myroides* infection is difficult because of extensive intrinsic resistance. The organism produces chromosomal beta-lactamases and efflux pumps that inactivate most beta-lactams and restrict the activity of other antibiotic classes. Even when in-vitro susceptibility is demonstrated, poor vascular perfusion and biofilm formation can limit clinical response [16,17]. In one prior report of carbapenem-resistant *Myroides* pneumonia, it was postulated that combination antibiotic regimens may be required [18]. In this case, infection progressed despite appropriate antibiotics in the form of carbapenem therapy and attempted revascularization, emphasizing the importance of early culture-guided treatment combined with decisive surgical management.

Amputation was ultimately required in our case for source control, which has been reported in other cases of diabetic patients with *Myroides* infection when conservative therapy failed [14,17]. Early recognition and multidisciplinary collaboration among vascular surgery, infectious disease, and wound-care specialists may improve outcomes, yet the combined effects of antimicrobial resistance and ischemia will always constrain therapeutic options.

## Conclusions

Clinicians should consider *Myroides* infection in both acute and chronic non-healing diabetic wounds that fail to improve with standard therapy, especially in patients with compatible risk factors to include exposure to water sources and hospital environments. Early culture acquisition, targeted antimicrobial selection, and timely surgical intervention remain central to effective management. Continued research is needed to define optimal antibiotic treatment strategies for this uncommon but clinically important pathogen, although early carbapenem therapy and potentially even combination antibiotic therapies should be considered.
